# Understanding patient and physician perceptions of benign prostatic hyperplasia in Europe: The Prostate Research on Behaviour and Education (PROBE) Survey

**DOI:** 10.1111/j.1742-1241.2007.01635.x

**Published:** 2008-01

**Authors:** M Emberton, M Marberger, J de la Rosette

**Affiliations:** 1 Institute of Urology and Nephrology, University College London London, UK; 2 Department of Urology, University of Vienna Vienna, Austria; 3 Department of Urology, AMC University Hospital Amsterdam, the Netherlands

## Abstract

**Aims:**

Benign prostatic hyperplasia (BPH) is a bothersome disease that can progress if left untreated. However, patient and urologist perspectives on BPH management are not fully understood. The aim of the Prostate Research on Behaviour and Education (PROBE) Survey was to assess healthcare-seeking behaviour and attitudes to BPH treatment in 502 BPH patients, and the beliefs and management practices of 100 urologists, from France, Germany, Italy, Spain and the UK.

**Results:**

The principal concerns of patients seeking medical advice were fear of cancer, sleep disruption, discomfort or embarrassment. The majority of BPH patients recalled receiving a digital rectal examination (61%), routine prostate-specific antigen (PSA) tests (67%) and prescription medication (72%). Eighty per cent of 5*α*-reductase inhibitor (5ARI) users vs. 68% of *α*-blocker users were satisfied with their treatment. More than half of the patients were concerned about requiring surgery or developing acute urinary retention, and > 75% would prefer a drug that provides reduction in the risk of surgery than one that provides rapid symptom relief. Most urologists performed digital rectal examinations (96%) and PSA tests (71%) on > 90% of patients presenting with BPH symptoms. Eighty-seven per cent of urologists believe that BPH progresses, and 78% believe that 5ARIs prevent BPH progression. However, most urologists prescribe *α*-blockers while few prescribe 5ARIs.

**Conclusions:**

This study highlights discrepancies between views and beliefs of patients and physicians regarding BPH and current practice in Europe.

**What's known**

- There is increasing recognition of the importance of the role of the patient in clinical decision-making and the importance of consideration of patient perceptions and preferences in ensuring selection of the appropriate management strategy and treatment success.
- A recently reported US national survey indicated that currently there are significant differences in the beliefs and attitudes of patients and physicians towards benign prostatic hyperplasia (BPH) (also known as enlarged prostate) and its management.

**What's new**

- This article provides information on the views and beliefs of both patients and urologists towards BPH and its management, and on current practice, across five European countries.
- This study confirms and extends the findings of the US survey and further highlights the need for improved communication between physicians and patients and greater involvement of the patient during clinical decision-making.

## Introduction

Benign prostatic hyperplasia (BPH) is a highly prevalent, bothersome disorder in ageing men; symptomatic BPH has been reported in approximately 25% of men over 40 years and more than 30% of men over 65 years (1). BPH can be defined histologically as a hyperplasia of the stromal and epithelial tissue of the prostate and it can be associated with lower urinary tract symptoms (LUTS), benign prostatic enlargement (BPE) and bladder outlet obstruction (BOO) (2,3). Patients with BPH experience a significant deterioration in quality of life because of their condition, reporting changes in sleep patterns, anxiety and embarrassment, altered mobility, changes in leisure, daily-living and sexual activities and in satisfaction with sexual relationships (4–6). In addition, BPH is progressive and can lead to worsening symptoms and the risk of serious long-term complications, such as acute urinary retention (AUR) and BPH-related surgery (7).

Traditionally, BPH treatment has focussed on achieving short-term relief of symptoms. While this remains a key management goal, there is a growing awareness that the progressive nature of the condition, coupled with patient perceptions and perspectives on treatment choices should inform management decisions (6). Current drug treatment choices for management of BPH include the commonly prescribed *α*-blockers and 5*α*-reductase inhibitors (5ARIs). The former class of drugs relaxes smooth muscle in the bladder neck and the prostate to provide rapid symptom relief, while the 5ARIs by inhibiting the conversion of testosterone to dihydrotestosterone (DHT) – the principal driver of prostate growth – provide long-term symptom relief and a reduction in the risk of AUR and BPH-related surgery (8–11).

Several recent surveys of patient attitudes to BPH and its treatment have revealed that men are concerned about disease progression – over 60% in one survey worried about the future need for surgery – and desire a drug therapy that will reduce the risks of complications and progression (12–14). Results from a US survey indicate significant differences between the views and attitudes of physicians and patients towards BPH and a lack of awareness among patients of the possible BPH therapies and their benefits (15).

This paper describes the findings of the Prostate Research on Behaviour and Education (PROBE) survey which was conducted in five European countries and sought the views of BPH patients currently receiving BPH therapy and practising urologists. The survey aimed to identify healthcare-seeking behaviour patterns in patients, assess the role of the healthcare provider in diagnosis and management, and evaluate attitudes to symptoms, complications and treatment of BPH.

## Methods

The PROBE survey was conducted in five European countries – France, Germany, Italy, Spain and the UK – over the period December 2002 to February 2003. The survey consisted of two questionnaires: one for patients with BPH who were receiving drug treatment for their condition, and the second for practising urologists. The patient and urologist questionnaires were executed by experienced researchers from the healthcare consultancy companies, Double Helix Development and Research International, respectively, and were designed to ensure answers were elicited without prompting.

### Patient survey

Recruitment of subjects for the survey was achieved through one of the following methods: physicians or nurses suggested participation in the survey to patients who met the inclusion criteria (below); or subjects were identified from screening surveys conducted on the street in which men were asked if they met the inclusion criteria. Physicians or nurses that recruited patients into the survey had been contacted by telephone, informed of the nature and needs of the survey, and were provided with details so that the patients could contact the recruiters.

Inclusion criteria for men entering the patient survey were: age 45–80 years; history of prostate problems; and BPH or an enlarged prostate leading the patient to consult with a physician in the past 12 months. To be eligible all subjects had to be receiving prescription medications for their prostate problem at the time of interview.

To ensure finasteride users were not over- or under-represented, recruitment to the survey was quota controlled such that 50% of respondents in each country were taking finasteride and the results of the survey were then weighted to reflect the national populations of finasteride users (national statistics for finasteride users were provided by GlaxoSmithKline). All responses were included in the analysis.

All eligible subjects took part in a 40-min, face-to-face interview based on a structured questionnaire divided into two parts. Part-1 of the questionnaire focussed on patients’ initial experiences relating to the diagnosis and management of BPH. This first part of the questionnaire involved 18 questions (some with subquestions) on topics, such as where patients first sought advice about their symptoms; the nature of presenting symptoms and the key reasons for seeking medical advice; the impact of initial symptoms on daily function and concerns held by patients at presentation; the process of diagnosis and the types of tests employed by the physician or urologist in reaching a diagnosis; information provided to patients on BPH at initial diagnosis; and the treatments and treatment information provided by healthcare providers in the initial stages of management. Part-2 of the questionnaire comprised a further nine questions (some with subquestions) relating to patients’ awareness and understanding of treatments prescribed for the management of BPH symptoms and disease progression. This section included questions relating to treatment outcome preferences, and was designed to assess the views and attitudes of patients to disease progression – specifically the development of AUR and the need for surgery.

### Urologist survey

Urologists in the five participating countries were identified through database searches and via hospital centres by an independent agency, Research International. Potential participants were approached by telephone or the internet and invited to take part in the survey, which was conducted by telephone interview and lasted approximately 15 min. All those who agreed to be interviewed received a stipend of 30–40 euros for their time and participation.

The urologist questionnaire comprised 23 separate items relating to physician views on the prevalence, diagnosis, prognosis and treatment of BPH; beliefs about patient perceptions of BPH; the treatment of BPH; and views on *α*-blockers and 5ARIs in BPH management. Questions were generally of a multichoice format, read out by the interviewer, with the option for the interviewee to decline to answer or to answer ‘do not know’ in most instances.

Collated data from both surveys are presented descriptively; no statistical analyses were performed.

## Results

### Patient survey

A total of 502 men – 100 from each of France, Italy, Spain and the UK and 102 from Germany – met the inclusion criteria and provided answers to the survey questionnaire.

#### Presenting symptoms and general awareness of BPH

The majority of respondents (85%) reported that their condition was discovered through symptoms rather than through routine examination by their healthcare provider. Of the 502 men surveyed, 69% of patients reported frequent micturition, 69% reported nocturia and 54% experienced a slower/weaker urinary stream as key presenting symptoms of their condition.

More than half of those surveyed (56%) stated that they felt ‘fairly’ or ‘very’ well-informed about health issues related to prostate problems, and in addition to consulting with their healthcare provider, 69% of patients reported that they had talked to their partner/spouse about their prostate problems.

#### Healthcare-seeking behaviour and main health concerns

On average, patients waited 10 weeks after they noticed symptoms before consulting their healthcare provider. Reasons cited by patients for delaying a consultation included a hope that the symptoms would go away or a belief that symptoms were an inevitable part of ageing.

**Figure 1:**
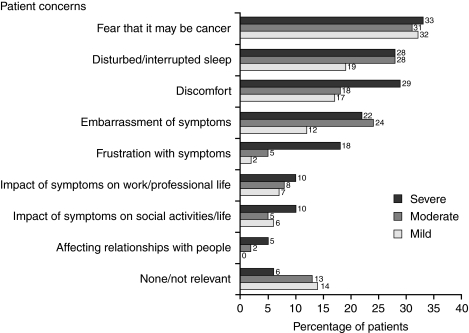
Proportion of patients with concerns (severe, moderate or mild) about their initial BPH symptoms (*n* = 502)

The main concerns first experienced by patients seeking medical advice for symptoms of BPH were the fear of cancer, disruption to sleep, discomfort and embarrassment (Figure 1). Almost one-third of subjects (32%) cited a fear of cancer as the reason for seeking medical help, and those with more severe symptoms, such as blood in urine, were more likely to harbour this underlying concern. The country-by-country variation in answers given by patients on healthcare-seeking behaviour was minimal (data not shown).

#### Diagnosis and monitoring

At first consultation, 61% of patients recalled having had a digital rectal examination (DRE), and 57% a prostate-specific antigen (PSA) test. Almost one-third of respondents (31%) reported that they received their diagnosis of BPH after one visit to their healthcare provider and 51% received a BPH diagnosis within two consultations. Sixty-seven per cent of subjects said that they routinely had PSA tests as part of their ongoing care.

#### Current symptoms

When asked about the ongoing symptoms of BPH experienced about half or more than half of the time in the past month, 41% of patients reported a weak urinary stream, 39% a sensation of not emptying their bladder completely, and 31% a need to push or strain to start urinating. A high proportion of patients (92%) reported getting up at least once in the night to urinate and within this group, 20% got up once, 35% twice, 19% three times, 8% four times and 10% five or more times in the night.

#### Drug treatment

Of all respondents, 72% were given a prescription medication for BPH symptoms on their first or second consultation. In response to questions relating to satisfaction with current therapy, more patients receiving the 5ARI treatment finasteride as monotherapy (80%) reported being ‘fairly’ or ‘very’ satisfied with treatment than patients on *α*-blocker monotherapy (68%) (Table 1). There was variation amongst countries: Italy had the greatest proportion of patients (85%) who were fairly or very satisfied with finasteride, while Germany had the lowest proportion (60%). This difference reflected the difference in the proportion of patients using finasteride in these countries.

**Table 1:** Patient satisfaction (very, fairly, not very, not at all) with *α*-blocker therapy alone (A) and 5*α*-reductase inhibitor therapy alone (B)

	Number of patients						
		
Patient opinion	UK	France	Germany	Italy	Spain	Total	Overall percentage of patients
**A**							
***α*-blocker**							
Not at all satisfied	3	0	0	0	0	3	1
Not very satisfied	5	5	10	6	2	28	10
Fairly satisfied	12	40	32	34	23	141	49
Very satisfied	16	17	4	4	14	55	19
Neither satisfied nor dis-satisfied	8	8	8	9	17	50	17
Do not know	3	2	4	0	2	11	4
Total	47	72	58	53	58	288	100
**B**							
**5*α*-reductase inhibitor**							
Not at all satisfied	0	0	0	0	0	0	0
Not very satisfied	1	0	1	2	0	4	6
Fairly satisfied	7	9	2	14	6	38	58
Very satisfied	5	1	1	3	4	14	22
Neither satisfied nor dis-satisfied	2	1	1	1	2	7	11
Do not know	1	1	0	0	0	2	3
Total	16	12	5	20	12	65	100

Nearly one-fifth of patients surveyed (19%) had their prescription medication changed since original diagnosis. The main reason cited for dis-satisfaction with current therapy was a lack of symptom improvement (38% of patients).

#### Complications associated with BPH

**Figure 2:**
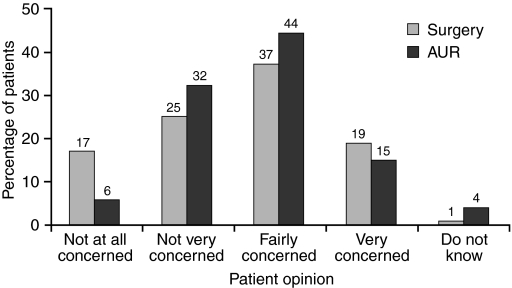
Patient concern (very, fairly, not very, not at all) about the requirement for surgery (total patient answers, *n* = 273) and the development of acute urinary retention (*n* = 117)

More than half of all respondents (*n* = 273, 54%) had discussed the topic of prostate-related surgery with their healthcare provider, and 56% of these patients reported that they were ‘fairly’ or ‘very’ concerned about surgery (Figure 2). Nearly a quarter of patients (*n* = 117, 24%) had discussed the issue of AUR, and 58% of these patients reported being ‘fairly’ or ‘very’ concerned about developing this complication (Figure 2). There was little country-by-country variation for patient concern with surgery and AUR (data not shown).

#### Views on treatment preference

**Figure 3:**
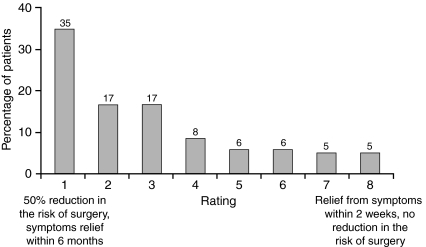
Patient selection of important drug attributes on a scale of 1–8, where 1 is a drug providing a 50% reduction in the risk of surgery and onset of symptom relief within 6 months, and 8 is a drug providing relief from symptoms within 2 weeks but no reduction in the risk of surgery (*n* = 502)

When asked to rate attributes of a drug treatment for BPH on a scale of 1–8, where 1 refers to a 50% reduction in the risk of surgery and onset of symptom relief within 6 months, and 8 refers to relief from symptoms within 2 weeks but no reduction in the risk of surgery, the average total score was 3.0. More than three-quarters of patients reported that they would prefer a drug that provides a 50% reduction in the risk of needing surgery (i.e. score 1–4) rather than a drug offering faster symptom relief (Figure 3). There was some country-by-country variation in patient treatment preferences; the proportion of patients giving a rating of 1 was highest in Spain (67%), lowest in the UK (23%). However, the overall view was in favour of reducing progression to surgery and this preference held true irrespective of whether the respondent was currently taking an *α*-blocker or a 5ARI-based therapy.

### Urologist survey

A total of 100 urologists – 20 from each of the five participating countries – took part in the survey. Of the urologist respondents, 85% see more than 10 new patients with symptomatic BPH every working month.

#### Beliefs about patient consulting behaviour

More than half of urologists (57%) considered that > 45% of the male population over 60 years of age are likely to suffer from BPH. Similarly, 60% believed > 60% of the male population over 80 years of age are likely to suffer from BPH.

In response to questions about why men with BPH symptoms may fail to seek medical advice, 45% of urologists stated the main reason to be that patients consider symptoms to be an inevitable part of ageing. Almost all urologists (91%) believed that patients with BPH eventually consult their physician because of bother from irritative or obstructive symptoms.

The majority of the urologists believed that it would be valuable (51% reported ‘very valuable’ and 40% reported ‘of some value’) to raise awareness of BPH in the general population within their respective countries.

#### Urologist assessment of BPH

The standard diagnostic assessments of a DRE and PSA assay were performed by 96% and 71% of urologists, respectively, on more than 90% of their patients presenting with symptoms suggestive of BPH. However, in the UK, only 35% of urologists performed a PSA test more than 90% of the time, making it an exception to the general practice across Europe.

#### Views on BPH progression and complications

When assessing patients with BPH during ongoing care, 56% of the urologists believed that a worsening of symptoms, the need for surgery, and AUR were all clear indicators of BPH progression. Indeed, 87% of the urologists believed that BPH is a condition that progresses. Yet only 58% of these same physicians believed that their patients understand that BPH can progress (Table 2). Country-by-country variation existed. The difference between the urologists’ own opinion on whether or not BPH progresses and the urologists’ perception of the patients’ opinion on this subject was greatest in Germany; 95% vs. 50%. The proportion of urologists believe that BPH progresses was lowest in the UK (70%). More urologists thought patients would progress to needing surgery than would progress to developing AUR. Greater than half of respondents (59%) believed that < 10% of their patients will progress to AUR in the next 4 years, while 82% believed > 10% of patients are likely to require prostate surgery in the next 4 years.

**Table 2:** Urologists’ opinion on whether BPH progresses (A) and on whether their patients realise that BPH progresses (B)

	Number of urologists					
		
Urologists’ opinion	UK	France	Germany	Italy	Spain	Overall percentage of urologists
**A**						
**Urologists’ opinion on whether BPH progresses**						
Yes	14	19	19	17	18	87
No	0	0	1	1	0	2
Some patients	6	1	0	2	2	11
Total	20	20	20	20	20	100
**B**						
**Urologists’ opinion on whether their patients believe BPH progresses**						
Yes	10	10	10	13	15	58
No	7	10	5	6	5	33
Do not know/refused	3	0	5	1	0	9
Total	20	20	20	20	20	100

#### BPH prescribing habits

Most urologists (63%) reported that they prescribe drug therapy to > 70% of their patients. Of the urologists who chose drug treatment, 58% prescribe *α*-blockers in > 70% of cases, while 5ARIs are used less frequently, being chosen by 47% of urologists for < 10% of cases (Table 3). The proportion of urologists prescribing *α*-blockers was highest in the UK (75% to > 70% of patients), whilst the proportion of urologists prescribing 5ARIs (45% to 31–70% of patients) was highest in Italy.

**Table 3:** Proportion of patients for which urologists prescribe *α*-blockers (A) and 5*α*-reductase inhibitors (B)

	Number of urologists					
		
Percentage of patients	UK	France	Germany	Italy	Spain	Overall percentage of urologists
**A**						
**Urologists prescribing*α*-blockers**						
< 10	0	0	0	0	0	0
10–30	1	0	1	0	0	2
31–50	0	1	6	0	1	8
51–70	4	7	6	9	6	32
> 70	15	12	7	11	13	58
Total	20	20	20	20	20	100
**B**						
**Urologists prescribing 5*α*-reductase inhibitors**						
< 10	11	11	16	3	6	47
10–30	7	7	4	8	12	38
31–50	1	2	0	6	2	11
51–70	1	0	0	3	0	4
> 70	0	0	0	0	0	0
Total	20	20	20	20	20	100

Among the factors considered when choosing to prescribe drugs were evidence of BPH progression (important to 66% of urologists) and prostate volume (important to 68% of urologists, although less of an influence on French urologists) (data not shown).

When asked which drug therapies they regarded as having potential to affect BPH progression, all Spanish urologists interviewed believed that 5ARIs are most effective in this regard, when compared with 60% of UK urologists. Although there was variation amongst countries, overall 78% believed 5ARIs inhibit BPH progression, while almost half (44%) thought that *α*-blockers may reduce the risk of disease progression (Table 4).

**Table 4:** Urologists’ opinion on whether *α*-blockers (A) or 5*α*-reductase inhibitors (B) are considered to reduce the risk of progression

	Number of urologists					
		
Urologists’ opinion	UK	France	Germany	Italy	Spain	Overall percentage of urologists
**A**						
***α*-blockers**						
Yes	9	9	8	11	7	44
No	7	10	12	9	13	51
Do not know	4	1	0	0	0	5
Total	20	20	20	20	20	100
**B**						
**5*α*-reductase inhibitors**						
Yes	14	16	16	12	20	78
No	4	3	3	6	0	16
Do not know	2	1	1	2	0	6
Total	20	20	20	20	20	100

## Discussion

The results of the PROBE survey confirm that most men initially consult their physician because of classic signs and symptoms of BPH related to LUTS, such as urinary hesitancy, frequent micturition and nocturia. There is little variation in healthcare-seeking behaviour between the European countries studied. In response to such symptoms, physicians appear to provide a rapid and thorough diagnostic work-up and instigate early use of drug treatment with a view to achieving fast and effective symptom relief. However, this survey also identifies that when patients and physicians are asked to consider the complications and progression of BPH, attitudes to treatment of the condition may vary between the physician and the patient. The PROBE survey indicates that over three-quarters of BPH patients would prefer a drug treatment that provides a 50% reduction in their risk of needing surgery in the future over a drug treatment designed principally for symptomatic relief. This is consistent with the results from US and British surveys which have demonstrated that patients are willing to trade immediate symptom relief for a reduction in the risk of progression (14,15). Indeed in the US survey, 73% of patients with mild symptoms of BPH and 75% of patients with moderate-to-severe symptoms rated a 50% decrease in the risk of surgery as an essential or important attribute for a new drug treating BPH; symptom relief within the first few weeks was selected as important or essential by < 60% of patients with symptoms of BPH (15). Thus, BPH patients may not be as concerned about rapid symptom relief as physicians have tended to believe.

The two main drug classes prescribed in daily management of BPH are *α*-blockers and 5ARIs. Both classes of drug provide symptomatic relief, with the *α*-blockers recognised to provide rapid symptom relief, and the 5ARIs known to reduce prostate size and reduce the long-term risk of BPH progression and its associated complications, such as AUR and BPH-related surgery (8–11). The PROBE survey suggests that, while as many as 66% of urologists profess to consider BPH progression when selecting a treatment and view 5ARIs as the treatment choice most likely to have an effect on disease progression, they employ 5ARI therapy in patient management less often than *α*-blockers. This disparity in beliefs and practices is at odds with patient preferences regarding the most important goals of BPH therapy.

Patient concerns and healthcare-seeking behaviour remained fairly consistent between countries. However, patient preferences with regard to BPH treatment did vary: more Spanish patients – and fewer UK patients – preferred a drug that reduces surgery over one that reduces symptoms. Urologist practices and beliefs also varied between countries: for example, UK urologists performed PSA testing much less frequently than other European countries. In addition, although overall most urologists believe that BPH progresses, the variation between countries ranged from 70% of urologists in the UK to 95% in France and Germany. There was also some country-by-country variation in the prescribing frequency of *α*-blockers and 5ARIs, with UK urologists being the highest prescribers of *α*-blockers. It seems likely that one of the underlying factors influencing both urologist and patient responses was the national healthcare systems. This could have been responsible for some of the country-by-country variation.

A number of recent surveys of patients with BPH have highlighted that patients with this condition do experience great worry over the prospect of surgery should their condition progress (12–15). The results of the PROBE survey suggest that if BPH patients were made aware of the relative benefits of *α*-blockers and 5ARIs, they may prefer to take a 5ARI to reduce their risk of surgery.

As patients become increasingly more involved in decisions about their healthcare, surveys gauging what matters to patients with chronic complaints in terms of clinical outcomes and health-related quality of life are becoming seen as more important. For example, a survey of women with stress incontinence (16) highlighted that more than three-quarters of women troubled by this common form of urinary incontinence are very bothered by their condition, and that often physicians fail to pick up on the aspects of urological complaints, which cause great stress to patients in their daily lives.

The PROBE survey highlights that BPH is a condition that concerns and worries patients despite receiving treatment. Early in the presentation, patients worry that their symptoms may indicate an underlying malignancy. When the disease is recognised as BPH, patients’ worry may then shift to a fear of the need for future surgery or the development of AUR.

This survey suffers from limitations inherent to all surveys, including the wording and order of questions and the potential influence and bias of the interviewer. In addition, only descriptive data are presented; statistical comparisons were not performed. However, this survey provides a valuable insight into the views and beliefs of patients and physicians regarding BPH and its management, and current practices across Europe and serves to highlight the need for further improvement in communication between physicians and patients and consideration of patient preferences during clinical decision-making.
